# Wearable and Implantable Intraocular Pressure Biosensors: Recent Progress and Future Prospects

**DOI:** 10.1002/advs.202002971

**Published:** 2021-01-21

**Authors:** Cheng Yang, Xinshuo Huang, Xiangling Li, Chengduan Yang, Tao Zhang, Qianni Wu, Dong liu, Haotian Lin, Weirong Chen, Ning Hu, Xi Xie

**Affiliations:** ^1^ State Key Laboratory of Optoelectronic Materials and Technologies Guangdong Province Key Laboratory of Display Material and Technology School of Electronics and Information Technology The First Affiliated Hospital of Sun Yat‐Sen University Sun Yat‐Sen University Guangzhou 510006 China; ^2^ School of Biomedical Engineering Sun Yat‐Sen University Guangzhou 510006 China; ^3^ State Key Laboratory of Ophthalmology Zhongshan Ophthalmic Center Sun Yat‐Sen University Guangzhou 510060 China

**Keywords:** biosensors, contact lens sensors, glaucoma, intraocular implantable sensors, IOP sensor

## Abstract

Biosensors worn on or implanted in eyes have been garnering substantial attention since being proven to be an effective means to acquire critical biomarkers for monitoring the states of ophthalmic disease, diabetes. Among these disorders, glaucoma, the second leading cause of blindness globally, usually results in irreversible blindness. Continuous intraocular pressure (IOP) monitoring is considered as an effective measure, which provides a comprehensive view of IOP changes that is beyond reach for the “snapshots” measurements by clinical tonometry. However, to satisfy the applications in ophthalmology, the development of IOP sensors are required to be prepared with biocompatible, miniature, transparent, wireless and battery‐free features, which are still challenging with many current fabrication processes. In this work, the recent advances in this field are reviewed by categorizing these devices into wearable and implantable IOP sensors. The materials and structures exploited for engineering these IOP devices are presented. Additionally, their working principle, performance, and the potential risk that materials and device architectures may pose to ocular tissue are discussed. This review should be valuable for preferable structure design, device fabrication, performance optimization, and reducing potential risk of these devices. It is significant for the development of future practical IOP sensors.

## Graphical abstract

Continuous intraocular pressure (IOP) monitoring, a promising measure for glaucoma's diagnosis and screening, provides a comprehensive view of IOP changes that is beyond reach for the “snapshots” measurements by clinical tonometry. This work summarizes materials, structures, working principles, performances, potential risks, and further applications of wearable and implantable IOP sensors. This discussion should be valuable for preferable IOP transducer's developments.

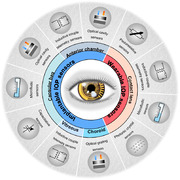

## 1 Introduction

Biosensors refer to the devices that record the information of life process by capturing biomarkers including IOP, heart rate, metabolites, bacteria, and hormones to offer valuable insights into the health of persons.^[^ ^1^ ^]^ With the rapid developments in nanomaterials, bioelectronics, and microfluidics, many unique properties have been endowed to wearable, implantable, and ingestible bio‐transducers.^[^ ^2^, ^3^ ^]^ For example, these wearable sensors maintain temperature,^[^ ^4^, ^5^ ^]^ and joints motion detection,^[^ ^6^, ^7^ ^]^ but also include additional capabilities in non‐invasive sampling of glucose,^[^ ^2^, ^8^, ^9^, ^10^, ^11^, ^12^, ^13^, ^14^, ^15^ ^]^ lactate,^[^ ^5^, ^16^ ^]^ cortisol,^[^ ^17^, ^18^ ^]^ and ions^[^ ^19^, ^20^ ^]^ from sweat and tears. Moreover, wearable transducers promise a practicable tactics to detect biological markers (uric acid,^[^ ^21^, ^22^ ^]^ pH,^[^ ^23^, ^24^ ^]^ enzymes,^[^ ^25^ ^]^ etc.) in wound bed for preferable wound care. Parallelly, biological sensors implanted in body provide direct solutions for tracking many vital information including intracranial pressure,^[^ ^3^, ^26^ ^]^ arterial‐pulse,^[^ ^27^ ^]^ neural activity,^[^ ^28^, ^29^ ^]^ and cellular electrical activities.^[^ ^30^ ^]^ Ingestible bio‐detectors is however equally important and highly long‐awaited since the devices allow straightforward sensing of pH, temperature, and gas within the gastrointestinal tract and its accessory organs.^[^ ^31^, ^32^, ^33^ ^]^ These devices have the potential to enable real‐time physiological monitoring and the early diagnosis of diseases and disorders. Among these apparatus, transducers worn on or implanted in eyes have achieved remarkable advances.^[^ ^11^, ^18^, ^34^, ^35^ ^]^ These sensors not only offers the abilities of direct monitoring of IOP,^[^ ^11^, ^36^ ^]^ and ion concentrations,^[^ ^20^ ^]^ humidity^[^ ^37^ ^]^ on ocular surface, but also bring the possibilities of registering glucose,^[^ ^11^, ^12^ ^]^ lactate,^[^ ^5^, ^38^ ^]^ and cortisol.^[^ ^18^ ^]^ Therefore, these detectors provide a promising methodology to record the critical physiological information and thus enable early screening and accurate diagnosis of ophthalmic disease, diabetes, liver diseases, etc. Especially for glaucoma, the typical ocular disease, these biological sensors have unique capabilities in real‐time monitoring of vital physical signs. It will provide more effective diagnostic basis and convenient access than the current clinic means.

### 1.1 Glaucoma

Glaucoma, the second leading cause of blindness globally,^[^ ^39^, ^40^ ^]^ has the terrible characteristic of irreversible and progressive vision loss.^[^ ^41^ ^]^ At present, there are no strategies to recover the already lost sight caused by the ocular disease. Earlier diagnosis combined with proper treatment is only optimal approach to slow the progression of visual loss caused by this ocular disorder.^[^ ^42^, ^43^, ^44^, ^45^ ^]^ However, screening and early diagnosis is still a significant challenge due to the slow and symptomless of the disease initially.^[^ ^11^, ^41^, ^44^ ^]^ Therefore, many patients with glaucoma are unaware of the disease's progression until it is severe.^[^ ^40^ ^]^ Although the eye is merely a small part of the body, it plays a very important role due to the indispensable visual sense in daily life.^[^ ^46^ ^]^ Hence, sight loss and vision impairment usually bring extensive troubles for a patient's daily life and also cause heavy burdens to the public medical service. Apart from irreversible blindness, typical glaucoma subtypes such as acute angle‐closure glaucoma are also accompanied by extremely painful symptoms.^[^ ^47^, ^48^ ^]^ It is therefore of great importance to better understand the reason of IOP elevations and the clinically available approaches of IOP examinations. Elevated IOP, the prime indicator for glaucoma diagnosis,^[^ ^45^, ^49^, ^50^, ^51^ ^]^ is attributed to abnormal circulation of aqueous humor.^[^ ^43^, ^45^ ^]^ The intraocular fluid is secreted by the ciliary epithelium, flows to the anterior chamber and finally drains via the trabecular meshwork from the eye.^[^ ^52^, ^53^ ^]^ If normal aqueous outflow cannot be maintained, the accumulated fluid inevitably causes IOP elevation that will arise ischaemic infarcts in retinal and optic nerve head lesions.^[^ ^43^, ^48^, ^53^ ^]^ Hence, apart from the optic nerve head and the retinal nerve fiber layer changes examination, IOP is regarded as an important indicative estimation factor for glaucoma diagnosis.^[^ ^35^, ^54^, ^55^, ^56^, ^57^ ^]^ Moreover, the treatment plan for glaucoma patients is mainly guided by the trend of measured IOP.^[^ ^58^ ^]^ Aside from glaucoma, many other diseases manifest causal or co‐occurrence relationship between increased IOP. However, the precise relationships between IOP and many diseases are still unclear.^[^ ^59^, ^60^, ^61^, ^62^ ^]^ Therefore, it is not be adequate to screening or accurate diagnosis most of related disorders by the value of IOP. These disorders^[63‐71]^ correlated with elevated IOP are summarized in **Table** **1**. Currently, many ophthalmotonometers have been applied in the ophthalmology department for IOP examinations, with each possessing their inherent strengths and weaknesses.

**Table 1:** List of diseases correlated with IOP

Symptoms/diseases	Description	Ref.
Traumatic brain injury, Brain tumor, Brain hernia	Elevated Intracranial (ICP) pressure is an important indicator for these disease diagnosis. Published works indicated that changes in ICP, at least in part, is relevant for IOP	^[^ ^63^, ^64^, ^65^, ^66^ ^]^
Graves' ophthalmopathy	Interactions between fibroblasts and T lymphocytes trigger tissue activations. The activated fibroblasts result in production of glycosaminoglycans and hyaluronan, which will press the eyeball inevitably. Accordingly, IOP exhibits pronounced correlations with clinical characteristics of Graves' ophthalmopathy	^[^ ^67^, ^68^ ^]^
Pseudoexfoliation syndrome	Pseudoexfoliation syndrome refer to an age‐related disorder of the extracellular matrix featured with progressive generation and excessive accumulation of a fibrillar substance in various ocular tissues.	^[^ ^69^, ^70^ ^]^
Chronic kidney disease	Published works have reported that the potential consequences of chronic renal failure on elevated intraocular pressure (IOP)	^[^ ^71^ ^]^

### 1.2 Conventional Approaches for IOP Measurement

At present, commonly clinical devices have been adopted for IOP measurement including Goldmann applanation tonometry, tonopen tonometry, pneumatonometer, and dynamic contour tonometry. Their characteristics are presented below.

Goldmann applanation tonometry (GAT) has been the gold standard for IOP examinations.^[^ ^72^ ^]^ In the IOP measurement process, a circular surface with 3.06 mm diameter of the corneal was applanated and then the required force will be shown accordingly.^[^ ^45^, ^73^, ^74^ ^]^ IOP is equivalent to the force divided by the flattened area according to the Imbert–Fick Law.^[^ ^75^ ^]^ This device could effectively enhance the IOP measurements precision (1 mmHg)^[^ ^76^ ^]^ and reduce the error derived from the rigidity of cornea.^[^ ^45^ ^]^ Therefore, GAT superseded earlier Maklakoff tonometry and Schiøtz tonometry in the IOP examination process.^[^ ^45^ ^]^ Both of the obsolete apparatuses are rarely applied in ophthalmic hospitals today already.^[^ ^45^ ^]^ Unfortunately, several inherent problems of the GAT contain i) local anesthetic and fluorescein must be performed before measurement. Notably, the amount of fluorescein in the tear film could also impact the accuracy of IOP examination by GAT. ii) It has to cooperate with the slit lamp, an inherently subjective process, during an IOP examination. iii) The Imbert–Fick Law is only suitable for tension‐free, thin membranes. Thus, the thickness and rigidity of cornea, as well as the existence of tear film will also introduce errors for IOP evaluation.^[^ ^45^, ^53^, ^77^, ^78^ ^]^

Tonopen is the most convenient and portable applanation tonometer.^[^ ^45^, ^53^ ^]^ However, calibration and local anesthesia must be carried out before the ophthalmic examinations. Furthermore, limited accuracy constraints the reliability of a single measurement by this equipment.^[^ ^78^, ^79^, ^80^ ^]^ Pneumatonometer, a non‐contact and continuous approach for the IOP examination, depends on airflow to flatten the cornea.^[^ ^81^, ^82^ ^]^ This method neither needs tedious pretreatment (local anesthesia and fluorescein) nor infection risk.^[^ ^45^ ^]^ However, the IOP examinations accuracy and reliability of the non‐contact tonometry are inferior to GAT^[^ ^83^ ^]^ and even to Tonopen tonometer.^[^ ^53^ ^]^ Dynamic contour tonometry could transform IOP into electrical signal by a piezoelectric module that needs to be pressed to the cornea with a minimal force.^[^ ^45^ ^]^ These apparatus allows continuous IOP measurement independent of central corneal thickness.^[^ ^77^ ^]^ However, preparations before measurement such as anesthesia are also needed.^[^ ^45^ ^]^

These apparatuses have been widely exploited in many hospitals worldwide. However, several considerable issues remain to be sorted out. i) Tonometry represents a kind of expensive equipment that needs many complicated preparations before IOP measurement. Therefore, the instrument is completely operated by a trained professional in the ophthalmic hospital at appointment time.^[^ ^42^, ^45^ ^]^ ii) IOP is commonly estimated by a single tonometric examination through clinical devices that is incapable of providing continuous IOP monitoring.^[^ ^84^ ^]^ Notably, IOP has been proved to fluctuate throughout the day in individuals suffering from glaucoma.^[^ ^85^, ^86^, ^87^, ^88^, ^89^, ^90^, ^91^, ^92^ ^]^ These significantly diurnal shifts are correlated with the worse condition of glaucoma.^[^ ^77^, ^88^, ^93^ ^]^ Thus, dangerous fluctuation in IOP and its rising above the safe range could inevitably be missed for weeks or months by the conventional ways for IOP measurements. The fatal omission usually causes inaccurate disease assessment, which will lead to the decision of unreasonable treatment options and even vision loss. Therefore, establishing continuous IOP examination strategies without temporal and spatial restriction is a central task for efficient screening, earlier diagnosis, optimized treatment scheme decision to glaucoma patients.

**Figure 1:**
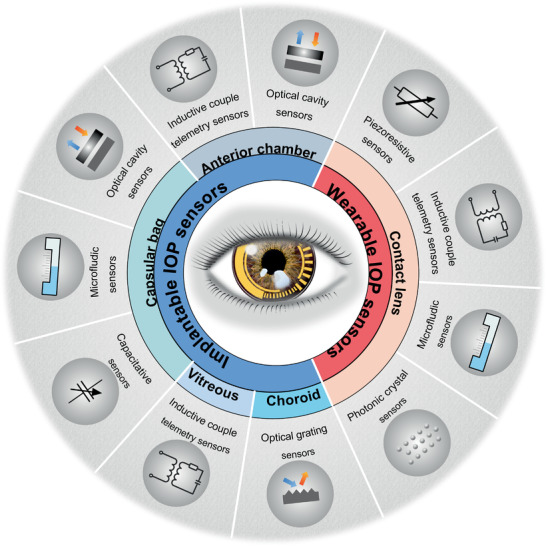
Schematic illustration of various pressure sensors for IOP monitoring. These state‐of ‐the art transducers include wearable IOP sensors (IOP sensors integrated on contact lens), and implantable IOP transducer (IOP sensors embedded in the anterior chamber, capsular bags, vitreous, and choroid.)

To this end, a great variety of techniques have been established for engineering wearable and implantable IOP sensors. The earlier device for continuous IOP monitoring was presented in 1967.^[^ ^94^ ^]^ This passive resonant‐based apparatus enables wirelessly continuous IOP observation, which provides an advanced strategy for IOP inspection. Then many efforts have been devoted to the development of wearable and implantable IOP examination devices. To date, significant research efforts have been offering substantial state‐of‐the‐art IOP biosensors based on inductive couple telemetry, piezoresistive, microfluidic, structural colors, and optical interference technologies. These devices provide promising approaches for in‐suit, continuous IOP monitoring. Previous reviews have reported many works about IOP monitoring devices.^[^ ^58^, ^95^, ^96^, ^97^, ^98^, ^99^ ^]^ Among these apparatuses, the devices‐based on contact lenses have been presented detailedly. However, to the best of our knowledge, a comprehensive review covering wearable and implantable IOP sensors reported recently is still rare. Herein, according to the ocular physiological structure, this review highlights the recent IOP monitoring advances and classified them into wearable IOP sensors (IOP sensors integrated on contact lens), and implantable IOP transducer (including IOP sensors embedded in the anterior chamber, capsular bags, vitreous, and choroid.) as illustrated in **Figure** **1**. Furthermore, their working principle, materials, designing and the potential risk that materials and device architectures may pose to interior and exterior tissue of the eye were discussed. This review would offer comprehensive summary of wearable and implantable biosensors for IOP continuous examination, and meanwhile provoke more significant efforts in this field.

## 2 Wearable IOP Biosensors

IOP caused by the dynamic balance of aqueous humor production and outflow in the anterior chamber was proven to acting on cornea certainly.^[^ ^45^, ^100^, ^101^ ^]^ Equally, the pressure could also lead to distortion of the contact lens shape.^[^ ^100^, ^101^, ^102^ ^]^ Hence the sensors fabricated on contact lenses are able to monitor the physiological change. Wearable IOP sensors are typically flexible devices‐based on contact lenses. Owing to the advantages of no infection risks and anxiety brought by surgery, the wearable transducers have been regarded as promising approaches for portable, non‐invasive, continuous, and in‐situ IOP monitoring. The earlier tonometer‐based on contact lenses was demonstrated in 1974.^[^ ^103^ ^]^ After several decades efforts from the academic and development community, advances in the wearable strategy for IOP monitoring have been substantial. Up to now, the wearable IOP sensors can be divided into piezoresistive, inductive couple telemetry, microfluidic and structural color sensors according to its operating principle.

### 2.1 Piezoresistive Sensors

Piezoresistive sensors, a kind of typical device‐based on the piezoresistive mechanism, exhibit changes in electrical resistance value when it is subjected to mechanical stimulus.^[^ ^106^ ^]^ Due to its simple fabrication process, uncomplicated structure, abundantly alternative materials, easy read‐out data and a broad range of pressure detection, these sensors were applied in IOP monitoring very early.^[^ ^102^, ^103^ ^]^ The factors causing piezoresistive properties in the field of IOP monitoring can be concluded as: 1) the changes in resistivity caused by rebuildable defects in active materials, and 2) caused by the deformations in geometry of the sensing element. The resistance (*R*) of a material can be expressed by the following equation:

(1) $$R=\frac{\rho l}{S}$$

where *ρ* represents the resistivity, *l* and *S* denote the length and cross‐sectional area, respectively.

**Figure 2:**
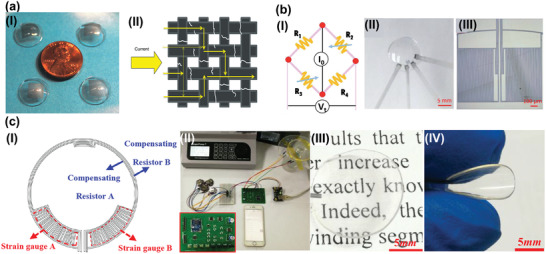
Wearable IOP sensors based on piezoresistive mechanisms. a) I) Image of the graphene woven fabrics‐based contact lens tonometer. II) The defects in graphene woven fabrics induced by IOP caused cornea deformation. Reproduced with permission.^[^ ^104^ ^]^ Copyright 2019, Springer Nature. b) I) Illustration of the Wheatstone bridge circuit. II) Photograph of the Wheatstone bridge circuit‐based wearable IOP sensors. III) Microscopy image of the strain gauge. Reproduced with permission.^[^ ^42^ ^]^ Copyright 2019, Royal Society of Chemistry. c) I) Schematic diagram of graphene‐based Wheatstone bridge circuit of the IOP sensor. II) Bluetooth module equipped wireless system for portable IOP measurement. III) Photograph of the graphene‐based IOP sensor with excellent transparency. IV) Optical image of the contact lens sensor with exceptional flexibility. Reproduced with permission.^[^ ^105^ ^]^ Copyright 2020, American Chemical Society.

For the piezoresistive mechanism induced by rebuildable defects in active materials, many cracks will be existing in active materials when IOP is increasing. These defects could reduce the carrier's mobility and result in lower *ρ* of the active material. For instance, Zhang and co‐workers have reported a low‐cost and lower power consumption contact lens tonometer‐based on graphene woven fabrics, as illustrated in **Figure** **2**a‐I.^[^ ^104^ ^]^ The graphene woven fabrics attached to the contact lens (24% water content) could exhibit high‐density cracks even in small eyeball deformation caused by IOP increasing. These defects, as described in Figure 2a‐II, could decrease the carrier's mobility (*μ*). With the decline of carrier's mobility, the *ρ* of graphene woven fabrics shows persistent growth according to the following relationship:^[^ ^107^, ^108^ ^]^

(2) $$\rho =\frac{1}{nq\mu }$$

where *n* refers to the carrier's concentration, *q* denotes the charge of an electron (1.602 × 10^−19^ C), respectively.

The crack propagation‐induced piezoresistive sensors have a common character of superior sensitivity due to that the gap can be rapidly rebuild even under minimal deformation.

Another strategy causing resistance change can be achieved by the clever structure design. These devices with specifically designed circuits undergo a change in the geometry of the sensing element when the IOP is elevating. Yu and co‐workers have presented a Wheatstone bridge circuit‐based non‐invasive IOP monitoring strategy (Figure 2b‐I) as depicted in Figure 2b‐II.^[^ ^42^ ^]^ The circuits were fabricated on PET substrate (thickness: 50 µm) by micro–nano processing. Then the flat device was thermally molded into contact lens shapes. The patterned metal electrodes deposited on PET substrate were served as a strain gauge (Figure 2b‐III) to detect the IOP changes. The ingenious circuit layout and design endow the device with outstanding sensitivity (20 µV mmHg^−1^) compared to other piezoresistive IOP monitoring sensors. These efforts have provided promising approaches to track the IOP fluctuation, yet the cables for power supply and data transforming will limit their long‐term applications, and hinder the wearer's activities. To accomplish portable IOP examination, a platform with Bluetooth module integrated on printed circuit board (PCB) was developed.^[^ ^105^ ^]^ The IOP variation could be sampled by graphene‐based Wheatstone bridge (Figure 2c‐I), then transmitted by Bluetooth wirelessly (Figure 2c‐II). The contact lens sensors comprised of few‐layer graphene enable wearable electronics with highly transparent (Figure 2c‐III), soft (Figure 2c‐IV), sensitivity, and biocompatibility for 24 h continuous IOP monitoring. However, further efforts should be devoted to minimizing the PCB circuits and to developing soft chip for ensure the practical application of the wearable system. **Table** **2** summarizes the sensing element materials, contact lens materials, and sensitivity of piezoresistive mechanism‐based wearable contact lens sensors for IOP monitoring.

**Table 2:** List of several wearable IOP sensors based on the piezoresistive mechanism and its sensitivity

Sensing element materials	Contact lens materials	Sensitivity	Ref
200 nm Pt/20 nm Ti	Silicone elastomeric layer (MED 6015)	8.37 µV mmHg^−1^	^[^ ^102^ ^]^
170 nm Pt ⁄25 nm Ti	Silicone elastomeric layer (MED 6015)	113 µV mmHg^−1^	^[^ ^109^ ^]^
*β*‐(ET)_2_I_3_	Hard contact lenses with XO2 material	1.5 Ω mmHg^−1^	^[^ ^110^ ^]^
50 nm Pt/10 nm Ti	PET	20 µV mmHg^−1^	^[^ ^42^ ^]^
Graphene	PDMS/Parylene/PDMS	150 µV mmHg^−1^	^[^ ^105^ ^]^

### 2.2 Inductive Couple Telemetry Sensors

**Figure 3:**
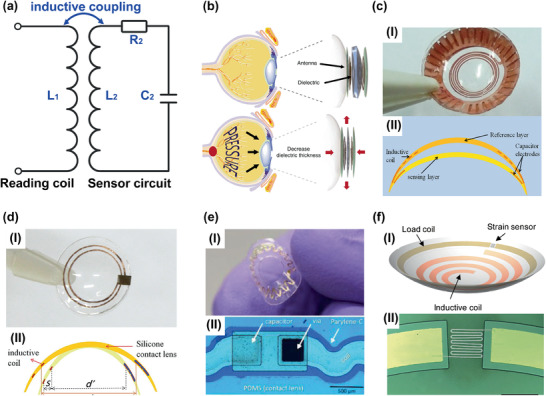
Inductive couple telemetry technology‐based wearable IOP sensors. a) The conceptual schematic of the inductive couple telemetry‐based IOP sensing system where L_2_, C_2_, and R_2_ are the inductance, capacitance, and resistance of the sensor circuit respectively. b) Graphene and Ag nanowires hybrid‐based contact lens IOP sensors and its sensing principle. Reproduced with permission.^[^ ^11^ ^]^ Copyright 2017, Springer Nature. c) I) The etched Cu foil electrodes‐base wearable IOP sensors. II) Structure of the inductive couple telemetry‐based contact lens sensor. Reproduced with permission.^[^ ^111^ ^]^ Copyright 2013, Elsevier. d) I) Thin film capacitor coupled with sensing coil‐based soft contact lens for tracking corneal curvature deformation. II) Schematic of the change in inductive coil diameter with contact lens curvature change. Reproduced with permission.^[^ ^112^ ^]^ Copyright 2014, Elsevier. e) I) A stretchable inductor‐based contact lens IOP sensors. II) Serpentine inductor and parylene C‐based sensor's circuit. Reproduced with permission.^[^ ^113^ ^]^ Copyright 2020, Royal Society of Chemistry. f) I) Strain sensor‐based wireless IOP sensing contact lens. II) The strain sensor integrated on the IOP sensor's circuit. Reproduced with permission.^[^ ^36^ ^]^ Copyright 2020, American Chemical Society.

Inductive couple telemetry sensors are the most frequently exploited devices for continuous, and non‐invasive IOP examination. The conceptual schematic of the inductive couple telemetry sensors‐based IOP monitoring system is shown in **Figure** **3**a. The wireless contact lens sensor features an inductor–capacitor–resistor (LCR) circuit with a corresponding resonant frequency (*f*):^[^ ^93^ ^][132]^

(3) $$f=\frac{1}{2\pi }\sqrt{\frac{1}{L_{2}C_{2}}-\frac{{R_{2}}^{2}}{{L_{2}}^{2}}}\approx \frac{1}{2\pi \sqrt{L_{2}C_{2}}}\;\mathrm{if}\;{R_{2}}^{2}<<\frac{L_{2}}{C_{2}}$$

where *L_2_*, *C_2_*, and *R_2_* refer to the inductance, capacitance, and resistance of the sensor, respectively.

The resonant frequency exhibits a shift when the value of either inductor or capacitor was altered by IOP induced corneal curvature changes. Sequentially, the resonant frequency shift was transmitted to an external reading coil by the inductive coupling link wirelessly. Therefore, IOP values could be accessed, which effectively avoids the damages and infection risk that cables result in cornea and conjunctiva. According to the different IOP sensing units, the inductive couple telemetry‐based wearable IOP sensors were subdivided into capacitive, inductive, and resistive types.

#### 2.2.1 Capacitive Sensing Units

The capacitance (*C*) of a parallel capacitor can be given as:

(4) $$C=\frac{\varepsilon_{0}\varepsilon_{r}S}{d}$$

where *ε*_0_, *ε*_r_, *S*, and *d* represent the space permittivity, relative permittivity, area, and the distance between electrodes, respectively. The changeable *d* is the main factor that has been applied to measure IOP. Specifically, IOP with the higher value could reduce *d* (the thickness of capacitor's dielectric layer), which will cause shift in resonant frequency value. The capacitive sensors are more appropriate for minimal force detection due to their high sensitivity to pressure changes and low power consumption.^[^ ^94^, ^114^, ^115^, ^116^, ^117^ ^]^ Therefore many research efforts tend to exploit it for the realization of inductive couple telemetry‐based wearable IOP sensors. For example, Kim and co‐workers have adopted graphene and its hybrid with Ag nanowires for contact lens IOP sensors preparation.^[^ ^11^ ^]^ The novel material offers sufficient transparency and flexibility for the detectors, which ensures excellent reliability, comfortability for wearers. To the realization of IOP sensing, Ecoflex was served as the dielectric layer. The flexible materials exhibit variable thickness during the IOP elevation (Figure 3b). The device wore on in‐vitro bovine eyeball exhibits perfect sensitivity (≈2.2 MHz mmHg^−1^). Dielectric layers with low moduli, undergo desired deformation when it is subjected to mechanical stimuli, are the key factor for engineering pressure sensor.^[^ ^116^ ^]^ While Ecoflex and other silicone elastomer featured with viscoelasticity and limited compressibility will bring the delayed response time and reduce the sensitivity.^[^ ^118^ ^]^ Air gap with smaller moduli, preferable viscoelasticity and compressibility represents another attractive alternative for fabricating capacitive pressure transducer. Chen and co‐workers have developed a wearable contact lens sensor for the IOP examination as shown in Figure 3c‐I.^[^ ^111^ ^]^ The capacitor electrodes and inductive coil were etched from Cu foil (thickness: 10 µm). These thick metal electrodes are facilitated to obtain higher quality factor (*Q*) for enhancing the sensors sensitivity.^[^ ^119^ ^]^ The structure of double‐layer contact lens was exploited to construct the air dielectric layer for the capacitor as shown in Figure 3c‐II. This clever design provides variable thickness for the dielectric layer that can be compressed by elevated IOP. However, it is a challenge to fabricate and assemble the capacitive sensors with specifically designed air chamber in a tiny contact lens.

Aside from the existing approaches (rubbers and air gap applications), other strategies exploited in tactile sensors to fabricate capacitive pressure transducers are highly worth learning to engineering IOP sensors. For instance, foamed silicone‐based dielectric layers offer ideal compressibility without complex fabrication process for building capacitive sensors.^[^ ^120^, ^121^, ^122^, ^123^, ^124^ ^]^ Further efforts also should be implemented to reduce the diameters of bubbles for acquiring thinner dielectric layers. It is of great important to achieve considerable capacitor's value and desirable signal‐to‐noise ratios.^[^ ^125^ ^]^ Microstructured elastomer dielectric represents another promising candidate to provide remarkable sensing performance for capacitive pressure transducers.^[^ ^27^, ^118^, ^126^, ^127^, ^128^ ^]^ The microstructured dielectric has a common pyramid arrays shape, which avoids the inherent viscoelastic behavior observed with bulk elastomers and shows extremely short relaxation times. Moreover, these microstructured dielectric layers can be fabricated by casting process meaning that their fabrication is scalable and relatively accessible.

#### 2.2.2 Inductive Sensing Units

For the sensors with variable inductance units, shapes of the inductive coil (outer diameter and turn spacing of the planar spiral inductances) could be changed by the curvature of cornea during IOP fluctuation. These parameter variations will result in alterations in the value of inductance,^[^ ^129^ ^]^ which cause the shift of resonant frequency accordingly. The variable inductor‐based pressure sensing technologies address the dielectric chamber leakage problems and undesirable baseline shifts normally seen in capacitive sensors.^[^ ^113^ ^]^ Moreover, inductive detectors are less susceptible to interference from the environment compared to the transducer‐based on capacitive, piezoelectric and piezoresistive mechanisms.^[^ ^130^, ^131^ ^]^ These strengths bring these sensors into sharp focus. For example, a contact lens IOP examination device composed of a thin film capacitor coupled with a sensing inductor was demonstrated as shown in Figure 3d‐I. As the IOP elevated, the corneal deformation could cause the diameter changes of inductor coil as shown in Figure 3d‐II. These changes in inductor size were always accompanied by inductance value alteration. While this design not be adequate to ensures apparent shape changes in inductance coil when IOP is elevation. Therefore, this wearable IOP sensor exhibits limited sensitivity. To overcome this limitation, proper design was employed. For instance, Kouhani et al. have presented a wearable IOP sensor (Figure 3e‐I) consist of a constant capacitor and a variable inductor that has a feature of stretchable serpentine wire.^[^ ^113^ ^]^ The inductor with a special layout was served as both the pressure sensor and the antenna as shown in Figure 3e‐II. Furthermore, the serpentine morphology inductor possesses preferable stretchability, which enhances the sensor's sensitivity. This effort provides a feasible strategy to develop a sensitive IOP monitoring system.

#### 2.2.3 Resistance Sensing Units

Apart from the shifts of resonant frequency, the return loss (S11) alterations could also be served as an important index to indicate the IOP changes. Specifically, the *R*_2_ in Figure 3a is a parasitic resistance with a constant value conventionally, which is generated from the circuit's electrodes. If a resistive strain sensor (*R*_s_) is exploited in series with C_2_ and L_2_, the inductance of the sensor circuit will be changed by the IOP fluctuations. Then the inductance matching between the reading coil and sensing circuit could be altered, which will exhibits variations in the value of S11. For example, a contact lens sensor with rigid‐soft hybrid structure was reported in 2019 as shown in Figure 3f‐I.^[^ ^36^ ^]^ The unique design was able to focus the strain produced by corneal deformation only in the resistive strain sensor (boron doped Si) shown in Figure 3f‐II. Therefore, the strain sensor could transmit the IOP into the value of resistance (*R*_s_). Accordingly, the performance of impedance matching between the reading coil and sensor circuit will be altered, which can change the S11 of the wireless system at constant resonant frequency. This work brings an innovative IOP monitoring system that allows noninvasive, and real‐time IOP measurements with desirable accuracy. However, it should be noted that boron doped Si, the materials of strain sensor adopted in this work, is a typical semiconductor. It can exhibit piezoresistive properties derived from band structure changes when it is loaded with strain or pressure.^[^ ^133^ ^]^ While the inherent charge transport behavior of the boron doped Si indicates that the resistivity of these strain sensors are susceptible to interference from environmental temperature.^[^ ^134^, ^135^ ^]^ To address this problem, Wheatstone bridge circuit is an attractive candidate to replace the Si‐based pressure sensor in future study due to their preferable sensitivity and extremely low temperature drift.^[^ ^42^, ^105^, ^136^, ^137^ ^]^ **Table** **3** summarizes the sensing mechanism, materials, and sensitivity of several inductive couple telemetry‐based contact lens sensors for IOP monitoring.

**Table 3:** Summary of the performance of inductive couple telemetry‐based contact lens IOP sensors reported in the literature

Sensing mechanism	Contact lens materials	Coil materials	Dielectric material	Sensitivity or minimum detectable IOP value	Ref
Capacitance	Commercialized soft contact lenses	Graphene and Ag nanowires hybrid	Ecoflex	About 2.2 MHz mmHg^−1^	^[^ ^11^ ^]^
Resistance	Silicone elastomeric layer (MED 6015)	Cr/Cu	NA	0.009 mmHg	^[^ ^36^ ^]^
Capacitance	Silicone rubber	Cu	Air	23 kHz mmHg^−1^	^[^ ^111^ ^]^
Inductor	Silicone rubber	Cu	Phthalocyanine	8 kHz mmHg^−1^	^[^ ^112^ ^]^
Inductor	NA	Ti/Au/Cu	Parylene C	57 kHz mmHg^−1^	^[^ ^132^ ^]^
Inductor	PDMS	Ti/Au/Cu	Parylene C	35.1 kHz mmHg^−1^	^[^ ^113^ ^]^

### 2.3 Microfluidic Sensors

**Figure 4:**
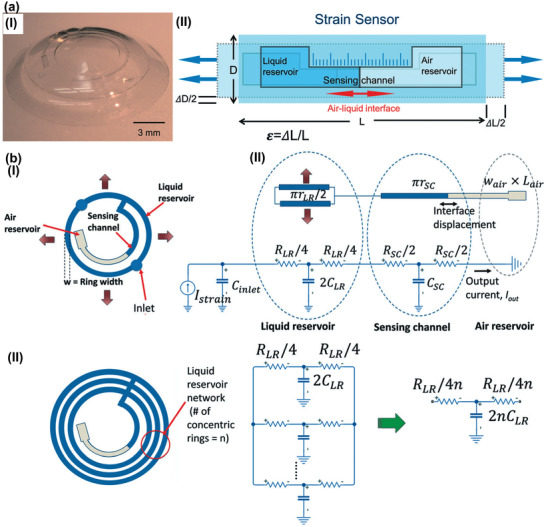
Wearable microfluidic IOP sensors. a) I) Image of the contact lens integrated with microfluidic strain sensor. II) The conceptual schematic of the microfluidic strain sensor operation principle.^[^ ^34^ ^]^ Copyright 2018, Royal Society of Chemistry. b) I) Schematic drawing of the microfluidic sensor design with a single liquid reservoir (left). The equivalent circuit model of the sensor (right). II) Schematic drawing of the microfluidic sensor design with multiple liquid reservoirs (left). The equivalent circuit model of the sensor (right). Reproduced with permission.^[^ ^138^ ^]^ Copyright 2019, Royal Society of Chemistry.

Wearable contact lens sensors‐based on microfluidic technology are emerging as a new methodology to track IOP fluctuation. The novel transducer, featuring excellent transparency, biocompatibility, and flexibility, could converts minimal IOP pressure signal into a huge fluidic volume expansion that can be observed and recorded directly by camera. For instance, a linear and stable response microfluidic IOP sensor was reported in 2018 as shown in **Figure** **4**a–I.^[^ ^34^ ^]^ The device is consists of a liquid reservoir and an air reservoir. The two chambers were connected by a sensing channel (Figure 4a‐II). The liquid reservoir volume was axially stretched by elevated IOP, which could cause the air‐liquid interface position toward the liquid reservoir. This change indicating IOP fluctuation could be captured by an external camera. Additionally, the work has summarized that the device's performance was critically affected by the absorption of the guide oil and the minuscule channel deformation under capillary forces. These factors inevitably cause drift, hysteresis, and instability for the microfluidics‐based pressure sensors. Overall, this work provides a new way to capture IOP fluctuations without integrating any electronic components. It is ideal for wearable contact lenses to reduce the complexity, cost, and potential risk of abrasion on eye tissue. To refine the performance of microfluidic IOP sensors‐based on contact lens, further work was reported by this group in 2019.^[^ ^138^ ^]^ They have built a computational model by equivalent circuit (Figure 4b) to promote the microfluidic sensor's designs and developments. In this work, microfluidic signal filtering in IOP monitoring was realized, which means that the noise induced by ocular pulsation and blinking can be suppressed without any aids of electronic components. This effort will dramatically boost the advancement of the microfluidic technologies‐based IOP transducers. Given these distinct features (visualized IOP changes and signal filtering function), the microfluidic IOP transducers seem to provide an approach that is closer to practical application for IOP monitoring. While there is still a greatest challenge to engineering portable systems matched with the microfluidic sensors for recording and sharing the long‐term IOP trends that is highly crucial for telemedicine.

### 2.4 Photonic Crystal‐Based Pressure Sensors

Photonic crystals, a kind of important nanoarchitecture, have a feature of spatially ordered morphology that reflects brilliant structural colors arising from the wavelength‐selective diffraction of light.^[^ ^139^, ^140^, ^141^ ^]^ The nanoarchitectures diffract light with specific wavelength according to Bragg's Law:^[^ ^142^, ^143^, ^144^ ^]^

**Figure 5:**
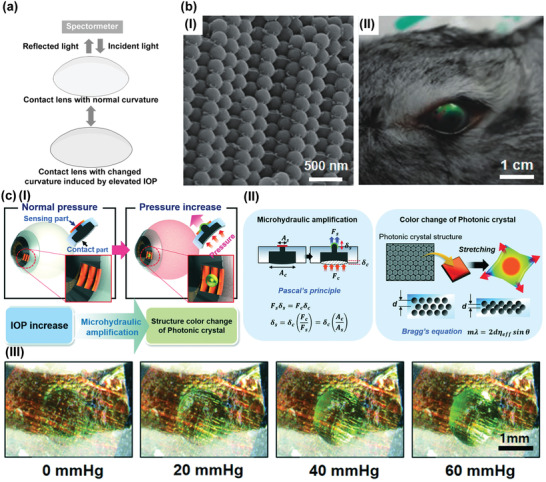
Photonic crystal‐based wearable IOP sensors. a) The conceptual schematic of the structural color‐based IOP sensor operation principle. b) I) SEM image of the periodic porous structures patterned by monodispersed silica colloidal particles. II) Photograph of structurally coloured contact lens sensor worn by a rabbit. Reproduced with permission.^[^ ^37^ ^]^ Copyright 2020, Royal Society of Chemistry. c) I) Illustration of the operating principles of the photonic crystal‐based contact lens sensor. II) The conceptual schematic of the microhydraulic amplification and the photonic crystal technology. III) Photographs of the color change of the photonic crystal‐based contact lens sensor according to the IOP changes of the eye model. Reproduced with permission.^[^ ^145^ ^]^ Copyright 2020, Royal Society of Chemistry.

(5) mλ=2ndsinθ

where *m*, *λ*, *n*, *d*, *θ*represent the order of diffraction, the wavelength of incident light, the mean refractive index of the system composed of colloids and voids, the spacing between the planes in the lattice, and the glancing angle between the incident light and diffraction crystal planes respectively. In the photonic crystal‐based pressure sensors, the lattice distance (*d*) will be changed by pressure. As a result, the reflected light shows a color shift from red to blue.^[^ ^145^ ^]^ Then the color changes could be recorded by spectrometer or even be perceived by naked eye, and camera theoretically when the structural colored strain sensors suffering enough pressure. For example, Song and co‐workers have reported an anodic aluminum oxide (AAO) film pattern‐base power‐free smart contact lens.^[^ ^146^ ^]^ Changes in contact lens curvature induced by elevated IOP will lead to spectrum shift alteration (**Figure** **5**a) that can be evaluated by a spectrometer. Results show that the sensor has a sensitivity of 0.02 nm mmHg^−1^. This advance provides a novel candidate for IOP measurements that bring a new research interest for pushing the development of this area. However, these wearable IOP sensors inspired by photonic crystals merely show limited wavelength shift in the reflectance spectra when the range of IOP is between 10 mmHg and 50 mmHg. It means that changes in these structurally colored contact lens sensors are extremely difficult to observe and identify by naked eye or smartphone camera. Furthermore, spectrometer represents a kind of expensive equipment that cannot offer portable measurement and uncomplicated operation. Therefore, developing more sensitive IOP contact lens sensors to provide unpowered and self‐reporting IOP monitored device is still attracting many researcher's interest. Wang and co‐workers have demonstrated silica colloidal particles patterned (Figure 5b‐I) structural color contact lens sensors for instrument‐free ophthalmic measurements.^[^ ^37^ ^]^ The device exhibits a perfect linear correlation between the wavelength of the reflectance peak of the structurally colored contact lens sensor and the pressure. Furthermore, the comfortability of the device was also examined in this work as shown in Figure 5b‐II. In this work, pHEMA hydrogel with water‐rich network structures were adopted to form contact lens. This means endows excellent biocompatibility and comfortability for the IOP sensors and paves the way for practical applications. To engineering a sensitive candidate for practically self‐reporting IOP measurement device, microhydraulic amplification technology was exploited to resolve IOP fluctuations with a naked eye.^[^ ^145^ ^]^ The deformation of the PDMS membrane integrated with photonic crystal is magnified by Pascal's principle in a microhydraulic system. Therefore, slight curvature variations of the cornea induced by elevated IOP could provide sufficient pressure to deflect the photonic crystal membrane intensively as illustrated in Figure 5c‐I. Then deformation of the membrane contributes to changes in lattice distance of the nanoarchitecture, which cause obvious color changes in the reflected wavelength as illustrated in Figure 5c‐II. The color changes could also be captured by a naked eye as demonstrated in Figure 5c‐III. The microhydraulic amplification technology‐based structurally colored contact lens sensor will bring a new way for instrument‐free IOP monitoring. Table 3, 4 summarizes the sensing mechanism and sensitivity of Photonic crystal sensors‐based contact lens sensors for IOP monitoring.

**Table 4:** Summary of the performance of photonic crystal‐based contact lens sensors for IOP monitoring

Sensing mechanism	Contact lens materials	Sensitivity (nm mmHg^−1^)	Ref.
Structural color	PDMS	0.02	^[^ ^146^ ^]^
Structural color	pHEMA	0.76	^[^ ^37^ ^]^
Structural color	Ostemer crystal clear 322	0.23	^[^ ^145^ ^]^

## 3 Implantable IOP Biosensors

Implantable biosensors are a kind of tool that can be embedded in the body for real‐time monitoring, and recording the critical physiological information of humans.^[^ ^147^ ^]^ These devices possess great potential to assessing health condition, providing quality care for individuals and also reducing budgets for the governments.^[^ ^148^ ^]^ Therefore, many efforts have been manage to engineering various implants to record arterial‐pulse,^[^ ^27^ ^]^ intracranial pressure,^[^ ^3^, ^149^ ^]^ pH,^[^ ^150^ ^]^ and glucose.^[^ ^151^ ^]^ Among these important signals, IOP that represents a primary factor indicating glaucoma has been attracted many focuses. And the sites have been chosen for implant IOP transducers include anterior chamber, capsular bags, vitreous, and choroid.

### 3.1 IOP Biosensors in Anterior Chamber

Anterior chamber, filling with aqueous humor, is the segment of the eyeball between the cornea and iris surface.^[^ ^152^, ^153^ ^]^ The IOP sensors implanted in the anterior chamber are immersed in aqueous fluid. Hence, the pressure caused by accumulated humor will exert on the devices, which manifested that the biosensors could register the IOP changes directly. Therefore, these implants can assess IOP values accurately without any cornea features interference such as rigidity, thickness that contact lens sensors and clinical tonometers confronted inevitably.^[^ ^77^, ^154^, ^155^ ^]^ Up to now, significant efforts have proposed many inventive devices‐based on electrical and optical technologies, which offer promising candidates for IOP monitoring and glaucoma diagnosis.

#### 3.1.1 Inductive Couple Telemetry Sensors

**Figure 6:**
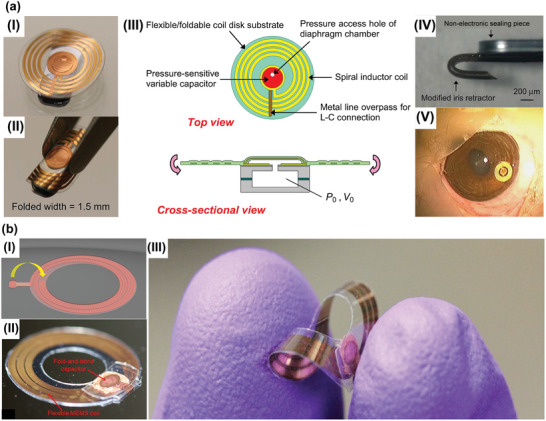
Inductive couple telemetry technology‐based IOP sensors implanted in anterior chamber. a) I) The photograph of the foldable pressure sensor. II) The photograph of the IOP sensor with folded state. III) Structure of the foldable pressure sensor. IV) The photograph of the anchor of the foldable IOP sensor for fixed on iris. V) The photograph of the foldable pressure sensor implanted in the anterior chamber of the rabbit eye. Reproduced with permission.^[^ ^156^ ^]^ Copyright 2010, The Institute of Electrical and Electronics Engineers, Incorporated (IEEE). b) I) The fold‐and‐bond technique. II) Full‐scale photographs of the passive pressure sensors. III) Excellent flexibility of the implantable IOP sensors. Reproduced with permission.^[^ ^157^ ^]^ Copyright 2013, The Institute of Electrical and Electronics Engineers, Incorporated (IEEE).

Implantable telemetric technology is one of the promising methods to realize continuous IOP examinations. The device was implanted in the anterior chamber for sensing IOP changes so that the important indicator can be wirelessly received by an external reading coil. These transducers with compact structure, low power consumption and cost enable straightforward IOP examinations, which have been attracted many focuses from 1967.^[^ ^94^ ^]^ However, existed IOP sensors implanted in anterior chamber have been confronted with a nonnegligible conflict between the device geometric size and the clinical requirement of minimally invasive surgery. Specifically, to achieve minimal painful and infection risk, the implantable device should be designed with a small size for minimally invasive operation. Conversely, to realize designed frequency, impedance matching, preferable coupling coefficients, quality factor and sensitivity, an inductance coil of implanted IOP sensors about a 4–10 mm diameter scale is required. To address this problem, a foldable pressure sensor featured with an LCR circuit was reported.^[^ ^156^ ^]^ The device fabricated on flexible parylene C substrate with a disk shape (diameter: 4 mm, thickness 1 mm, shown in **Figure** **6**a‐I) and can be folded into minimal size (4 mm × 1.5 mm × 1 mm, shown in Figure 6a‐II). Facilitate by the gas cavity structure (shown in Figure 6a‐III), the capacitive sensor has a pressure reference in IOP examinations. Moreover, a hook (exhibited in Figure 6a‐IV) of the device penetrates the iris stratum to locate the device in the anterior chamber as demonstrated in Figure 6a‐V. To form the capacitor cavity, complicated multiple overlay exposures were implemented, which is tedious and time‐consuming. To simplify the fabrication process, a fold‐and‐bond technique was adopted to form an LCR circuit by integrated planar MEMS coil and capacitor's panels as illustrated in Figure 6b‐I. The foldable capacitor's panel combined with rigid SU‐8 sidewalls were adopted to build a gas chamber.^[^ ^77^ ^]^ The photograph of this passive device was exhibited in Figure 6b‐II.^[^ ^157^ ^]^ The excellent flexibility of the implantable was verified by Figure 6b‐III.^[^ ^77^ ^]^^[157]^ The pressure induced panel deformation will cause chamber volume changes that could alter the value of capacitor and shift the resonant frequency of the LCR sensors. These works provide excellent solutions to address the challenges in surgery and fabrication process of implantable telemetric IOP sensors, which will accelerate the development of implantable IOP monitoring approaches. **Table** **5** summarizes the sensing mechanism, materials, size, and sensitivity for several inductive couple telemetry‐based implantable IOP sensors.

**Table 5:** Summary of the sensing mechanism, materials, size, and performance of inductive couple telemetry‐based sensors for IOP monitoring^[^ ^77^, ^93^, ^156^, ^157^, ^158^, ^159^, ^160^ ^]^

Sensing mechanism	Substrate materials	Coil materials	Device size	Sensitivity	Ref
Capacitance	Parylene C/Si	Ti/Au	4 mm × 1.5 mm × 1 mm (foldable)	243 kHz mmHg^−1^	^[^ ^156^ ^]^
Capacitance	Parylene C/Si	Ti/Au	4 mm × 1 mm	1.14 MHz mmHg^−1^	^[^ ^93^ ^]^
Capacitance	PI	Cu	Diameter: 12 mm	119.88 kHz mmHg^−1^	^[^ ^158^ ^]^
Capacitance	Glass/Silicon	Au	2.6 mm × 1.6 mm × 1 mm	120 kHz mmHg^−1^	^[^ ^159^ ^]^
Capacitance	SiO_2_/Silicon	Al	Thickness: 560 µm	1.5 kHz mmHg^−1^	^[^ ^160^ ^]^
Capacitance	Parylene C	Ti/Au	Diameter: 14 mm Thickness: 85 µm	156 kHz mmHg^−1^	^[^ ^77^, ^157^ ^]^

#### 3.1.2 Optical Cavity‐Based Pressure Sensors

**Figure 7:**
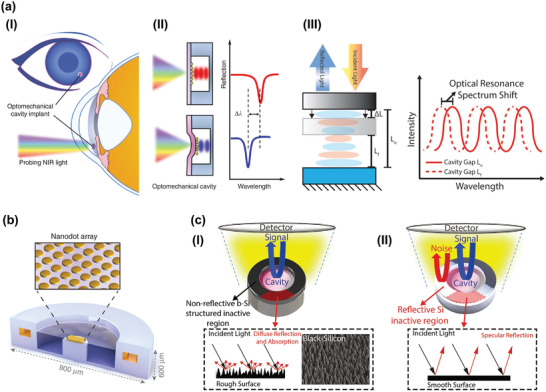
Optical cavity‐based IOP sensors implanted in the anterior chamber. a) I) IOP sensor implanted in the anterior chamber for IOP examination with NIR light. II) A schematic of the shift in the optical resonance spectrum when the top plate exhibits deform induced by elevated IOP. Reproduced with permission.^[^ ^161^ ^]^ Copyright 2017, Springer Nature. III) A schematic of the optical resonance spectrum shift when the top membrane exhibits movement induced by elevated IOP. Reproduced with permission.^[^ ^162^ ^]^ Copyright 2017, Wiley VCH. b) The structure of nanodot‐enhanced implantable sensor. Reproduced with permission.^[^ ^161^ ^]^ Copyright 2017, Springer Nature. c) I) The Si‐only sensors with higher noise. II) The sensor integrated with black Si exhibits a higher signal‐to‐noise ratio. Reproduced with permission.^[^ ^162^ ^]^ Copyright 2017, Springer Nature.

Cornea and aqueous humor represent natural body window^[^ ^36^ ^]^ with thin thickness and limited near‐infrared (NIR) light absorption.^[^ ^161^ ^]^ Given these critical reasons, significant efforts have sought to implant minimal optical cavity‐based sensors in the anterior chamber for IOP monitoring as shown in **Figure** **7**a‐I.^[^ ^161^ ^]^ This sensor mainly consists of a bottom fixed reflective surface and a top deformable or movable semitransparent plate, which form an optical cavity with an air gap. The elevated IOP induced pressure difference between the inner optical cavity and anterior chamber could deflects^[^ ^161^ ^]^ (Figure 7a‐II) or moves^[^ ^162^ ^]^ (Figure 7a‐III) the top semitransparent plate gradually. Accordingly, the air gap thickness of the cavity will be changed, which results in a shift in the reflected resonance spectrum derived from thin film interference principle.^[^ ^163^, ^164^ ^]^ The changes correlated with IOP is captured by a spectrometer as demonstrated in Figure 7a‐II,III. For example, a sub‐1 mm IOP sensing implant was reported in 2016.^[^ ^165^ ^]^ The rough silicon surface in the surrounding area was adopted as an antireflection structure to reduce background noises. Gold nanodot array with the area of 200 × 200 µm was deposited on the silicon‐nitride membrane for enhancing the NIR reflectivity. The strategy based on thin film interference principle offered an implant with compact size and accurate IOP measurements at a practical readout distance. To achieve better sensitivity, a nanodot‐enhanced implantable sensor (diameter: 900 µm; thickness: 600 µm) was fabricated by this group demonstrated in Figure 7b.^[^ ^161^ ^]^ In this paper, the group has summarized critical factors for the design of optical cavity sensors including NIR wavelength, dimensions of a gold‐nanodot array, deformable membrane and its diameter as well as thickness. This work provides a passive IOP measurement system with high resolution, and low risks in implantation procedures. While further efforts should be made to decrease background noise detection, which is highly meaningful for practical IOP sensing. To effectively enhance the signal‐to‐noise ratio (SNR) of the optical sensors, antireflective black Si was integrated into the inactive region of the sensor (Figure 7c‐I).^[^ ^162^ ^]^ The improved device exhibited root‐mean‐square error of 0.58 mmHg and peak‐to‐peak variation <±2 mmHg, which is superior to the Si‐only device (root‐mean‐square error: 1.96 mmHg, peak‐to‐peak variation: ±8 mmHg) as shown in Figure 7c‐II. These passive sensors maintain a small size, but also include additional capabilities of the higher signal‐to‐noise ratio. However, it is noted that near‐infrared (NIR) light source, microscope, spectrometer, and slit‐lamp were indispensable to measure IOP by these means.^[^ ^161^, ^162^ ^]^ Although great challenges limit the practical applications for the aim of portable and continuous IOP measurements by these optical sensors, this trend shows the attention of research on IOP monitoring devices toward differentiation and precision.

### 3.2 IOP Biosensors in Capsular Bags

Capsular bag is a chamber that generated by capsulorhexis and lens extraction in the cataract surgery process.^[^ ^166^, ^167^ ^]^ This bag, consists of a portion of the anterior capsule and the entire posterior capsule, was built for housing intraocular lens (IOL) and its appurtenance to address cataract disease.^[^ ^166^, ^168^ ^]^ Due to the mature cataract surgery with minimal invasion and low infection risk, the site has been selected by many efforts to implant IOP sensors that are integrated on IOL or capsular tension ring. In this section, we present an overview of the latest advances about IOP transducers implanted in capsular bags.

#### 3.2.1 Capacitive Sensors

**Figure 8:**
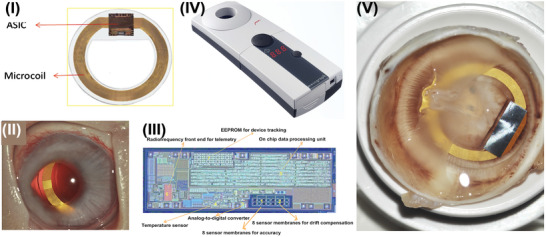
Capacitative IOP sensors implanted in the capsular bags. I) Image of the ring‐shaped IOP sensors; II) In vivo photograph of the transducer placed within the capsule bag; III) Inside view of the ASIC; IV) Picture of the external IOP reading device; V) Risk assessment for long‐term implantation of IOP detector. Reproduced with permission.^[^ ^170^ ^]^ Copyright 2011, Association for Research in Vision and Ophthalmology (ARVO).

Capacitive pressure sensor, a device translating pressure changes into capacitance variations, is a kind of typical transducer with many strengths.^[^ ^169^ ^]^ In the area of implantable IOP measurement, capacitive IOP sensors could be embedded in capsular bags by clinical cataract surgery. This strategy, suitable for the realization of microincision and low infection risk, is highly regarded by the research community. For instance, an intraocular monitoring device featured with ring shapes (**Figure** **8**‐I) that was implanted within the lens capsule (Figure 8‐II) was reported in 2011.^[^ ^170^ ^]^ An array of capacitive pressure sensors was integrated into application‐specific integrated circuit (ASIC) as exhibited in Figure 8‐III. Additionally, a micro‐coil (outside diameter:11.3 mm, inside diameter: 7 mm, thickness:0.9 mm, weight: 0.1 g) was connected with the ASIC for power supply and data transmission. Elevated IOP will deflect the membrane of capacitor, then the change was detected by external reading device as shown in Figure 8‐IV. Animal experimentations demonstrated that long‐term implantation of IOP detector will not produce fibrotic membranes (as presented in Figure 8‐V) and even inflammation. The implant offers an innovative approach that allows continuous IOP sampling without cornea contacting. This strategy is especially appropriate for the glaucoma patients who are also suffering from cornea disease or who has treated by keratoplasty. However, the telemetry technology‐based wireless IOP examination usually suffer from limited transmission distance. To meet actual demands, a capsular tension ring structured IOP sensor was developed and reported in 2014.^[^ ^171^ ^]^ The device consists of coil, RF chip and a capacitive pressure sensor could be implanted in capsular bag by cataract surgery. It could expand the capsular bag, stabilize intraocular lens and also detect the IOP changes. Specifically, the IOP induced capacitance changes are converted to frequency value through RF chip. Power and data transmission were achieved by coil antenna. This strategy is supposed to bypass the limited communication range, which provides new approach for the practical application of implantable IOP monitoring devices.

#### 3.2.2 Microfluidic Sensors

**Figure 9:**
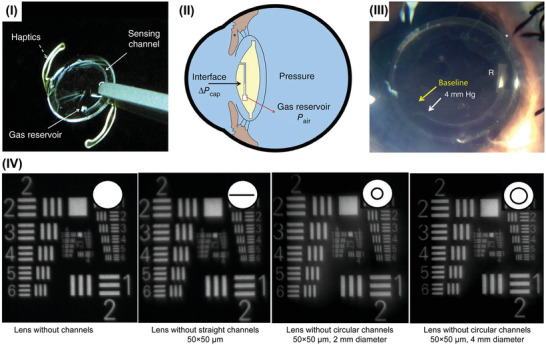
Microfluidic principle‐based IOP sensors implanted in the capsular bags. I) Photograph of the microfluidic pressure sensor embedded within an intraocular lens; II) Schematic illustration of the structure and working principle of the microfluidic pressure sensor; III) Microfluidic sensor implanted in porcine eyes. IV) Optical performance of the implantable device. Reproduced with permission.^[^ ^35^ ^]^ Copyright 2014, Springer Nature.

Microfluidic IOP sensors have inspired many research efforts to explore their application in wearable IOP monitoring. That device with a simple structure, low cost provides a promising approach that enables IOP to be read with a cellphone camera or naked eye. In addition to the flexible contact lens, intraocular lens (IOL) is another ideal platform for microfluidic channels fabrication and long‐term IOP examinations. A microfluidic principle‐based implantable IOP sensor was reported in 2014 as demonstrated in **Figure** **9**I.^[^ ^35^ ^]^ The special IOL was designed with an interior gas reservoir that connected to the aqueous intraocular liquid by airtight microfluidic channel. Liquid was driven into channel by capillary forces and IOP, which forms a gas‐liquid interface in the microfluidic channel as exhibited in Figure 9II. Elevated IOP provokes the interface to shift toward the gas reservoir as shown in Figure 9III. Conversely reduced IOP causes a move toward the channel opening. These changes allow direct reading by a camera or naked eye. Therefore, the IOP fluctuations were analyzed by the image of aqueous‐air interface position. The passive implant shows excellent sensitivity (smaller than 1 mm Hg), stability and linear relation between IOP and interface position. Moreover, the sensitivity of this device could be refined by elevating the ratio of reservoir volume to channel cross‐section, which is relatively accessible. While the special intraocular lens integrated with microchannels, the device still exhibits excellent optical performance without significant optical aberrations as shown in Figure 9IV. This work demonstrated a passive IOP monitoring transducer that possesses self‐reporting capability and allows home base examinations. Therefore, the effort provides a valuable device that has the ability to provide quality care for glaucoma patients.

### 3.3 Optical Cavity‐Based Pressure Sensors

**Figure 10:**
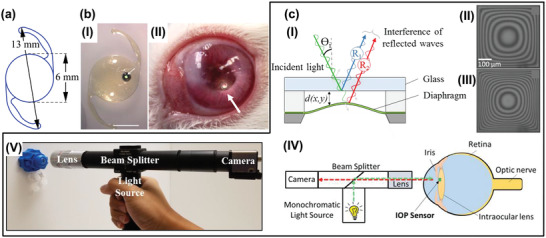
Optical IOP sensors implanted in the capsular bags. a) The physical dimension of IOL. b) I) Optical microcavity IOP sensor attached on IOL. II) The optical sensor implanted in a rabbit eye. Reproduced with permission.^[^ ^172^ ^]^ Copyright 2016, Association for Research in Vision and Ophthalmology (ARVO). c) I) Schematic illustration of light reflections at the sensor cavity. II) Images of interference patterns observed from an in‐vitro experiment at the pressure of 0 mmHg and III) 40 mmHg. IV) Schematic and V) photograph of the portable reader for the optical intraocular pressure measurement system. Reproduced with permission.^[^ ^173^ ^]^ Copyright 2018, The Institute of Electrical and Electronics Engineers, Incorporated (IEEE).

IOL with minimal size is a clever design of implants to the realization of microincision techniques in cataract surgery. However, it is difficult to realize complete structure, and also maintain unobstructed view in the limited space (**Figure** **10**a‐I) for many strategies that were performed to engineering novel IOL for IOP monitoring. Optical cavity‐based pressure sensors featuring small size and higher signal‐to‐noise ratio provide a unique approach to address this problem. Research efforts have been sought to develop the optical sensors equipped IOL that were implanted in capsular bags for long‐term IOP monitoring. For instance, Lee and co‐workers have reported a microcavity (800‐micron‐diameter) integrated IOL (Figure 10b‐I).^[^ ^172^ ^]^ Results show that the implant exhibited excellent SNR (13 dB) in vivo measurements (Figure 10b‐II). This development has facilitated the advancement of implantable IOP sensors, avoiding vision obstructing and further difficulty for implant process. However, the utilization of NIR light source and spectrometer limits the feasibility of this optical sensors inspired home‐base IOP examinations. To bypass the use of complicated instruments for collect the cavity sensors reported signal, an interferometric pressure sensor was reported.^[^ ^173^ ^]^ The device consists of silicon nitride film (thickness: 200 nm), SU‐8 spacer (thickness: 10 µm) and glass substrate (thickness: 200 µm). The reflected ray of silicon nitride film surface and glass substrate surface could form stable interference fringes when the sensor was illuminated by monochromatic light as demonstrated in Figure 10c‐I. The deformation of silicon nitride film caused by pressure will alter the optical path difference of reflected ray that results in the changes of interference fringes inevitably as shown in Figure 10c‐II,III. Accordingly, the phenomenon was able to capture by a portable reader (Figure 10c‐IV,V) to facilitate the analysis of IOP fluctuation. This system equipped with handheld reader provides a viable means for IOP examination with a smartphone, which has advanced the optical cavity‐based IOP sensors for clinical applications.

### 3.4 IOP Biosensors in Vitreous

**Figure 11:**
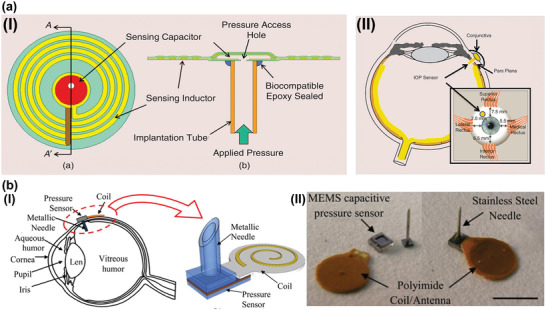
Inductive couple telemetry technology‐based IOP sensors inserted in the vitreous cavity. a) I) Top view of the sensing capacitor and coil (left) and cross‐section of the IOP transducer (right). (II) Schematic exhibition of the implant site of the IOP sensors featured with tube. Reproduced with permission.^[^ ^174^ ^]^ Copyright 2012, The Institute of Electrical and Electronics Engineers, Incorporated (IEEE). b) I) Schematic of the implant site and structure of the IOP sensors featured with needle. II) Photograph of the IOP sensors featured with needle. Reproduced with permission.^[^ ^176^ ^]^ Copyright 2013, The Institute of Electrical and Electronics Engineers, Incorporated (IEEE).

The vitreous represents the largest ocular part with semisolid structure that fills the space in the center of the eye.^[^ ^152^ ^]^ The transparent viscoelastic gel could be act as a channel for conduct the IOP generated in anterior chamber to retina. Therefore, vitreous has been considered as another ideal site for embedded IOP sensors to accomplish long‐term IOP monitoring by researchers. Currently, the reported IOP sensors inserted in vitreous were mainly‐based on the technology of inductive couple telemetry. It commonly has features of sensing capacitor and needle with higher length‐diameter ratio. For instance, IOP sensor assembled with capillary tube was developed.^[^ ^174^ ^]^ The LCR circuit comprised of captative pressure sensor and coil were located outside of eyeball with access to sensing the IOP by hypodermic needle or capillary tube (**Figure** **11**a‐I) penetrate into the vitreous body shown in Figure 11a‐II. The design ensures direct IOP examinations with simple implantation surgery. The strategy proposed a new concept and approach about implantable IOP sensors that enables continuous and wireless IOP measurements without complicated surgical procedure. To approach scalable and relatively accessible fabrication process, hypodermic needle (30 gauge) was exploited to transport the IOP to pressure sensor as illustrated in Figure 11b.^[176]^ The IOP fluctuates could be detected by pressure sensor and transmitted to external reading coil in the forms of resonant frequency. These schemes that enables direct hydraulic connection with the vitreous bring unique solution for IOP measurement with simple invasive operation. However, several inherent problems also remained. For instance, Carrasco reported a less invasive IOP system consist of pressure sensor and capillary tube.^[^ ^175^ ^]^ In this paper, they pointed that intraocular fibrosis, the interference of tear film, eyelid acting, and potential irritations that minimal invasive device caused should be consider in further study.

### 3.5 IOP Sensors on Choroid

**Figure 12:**
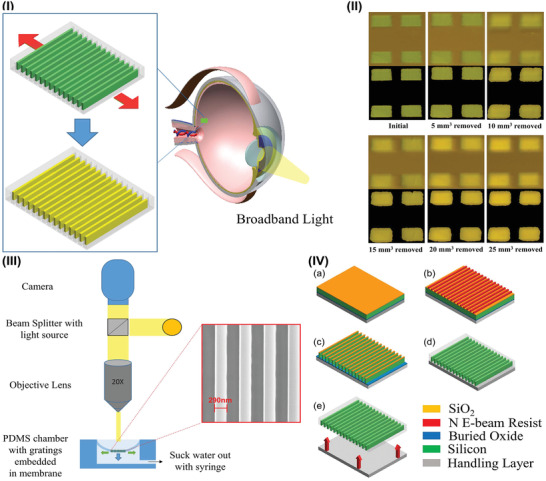
Grating technology‐based IOP sensors implanted on the choroid. I) Illustration of the optical sensor's working principle. II) Changes in the observed color of grating technology‐based implants. III) Schematic illustration of the characterization approaches. IV) The fabrication process of optical grating for IOP monitoring: First, SiO_2_ film was deposited on Silicon‐On‐Insulator wafer by PECVD. Then, the SiO_2_ film was patterned by E‐beam lithography, development and RIE to forming the hard mask and silicon gratings. Sequently, buffered oxide etch was used to etch the SiO_2_ grating mask and made an undercut in the buried oxide layer below the silicon gratings array to facilitate the transfer printing process. Finally, PDMS was spin coated and cured on top of the sensor for transfering process. Reproduced with permission.^[^ ^178^ ^]^ Copyright 2018, The Institute of Electrical and Electronics Engineers, Incorporated (IEEE).

The choroid refers to a thin, highly pigmented, vascular ocular tissue located between the retina and the sclera.^[^ ^152^, ^177^ ^]^ This tissue has capabilities in nourishing the retina, absorbing unexpected light for enhancing quality of vision.^[^ ^152^ ^]^ In the research field of implantable IOP sensors, this ocular part was selected as a site for embed strain sensors for IOP monitoring. For instance, optical grating technology was adopted to develop an implant for IOP examination as exhibited in **Figure** **12**I.^[^ ^178^ ^]^ The device, sited on choroid in ocular, is a near‐wavelength high contrast grating array. IOP fluctuation could induce the change in the grating period that results in a variation in the observed color (Figure 12II) that can be recorded by camera through microscope lamp and objective lens (Figure 12III). This strategy enables us to measure minimal deformations according to Bragg's Law.^[^ ^142^, ^143^ ^]^ The implantable biosensors could be fabricated by micro‐nano‐fabrication process (Figure 12IV), which is relatively accessible. This work offers a unique approach to detect the IOP changes, which indicates that the developments of implantable IOP transducer are moving towards innovation and differentiation.

## 4 Current Status and Limitations

In this review, we presented the severity of glaucoma, clinically available IOP examinations for diagnosis of glaucoma and its limitations. Additionally, the main progress of IOP sensors in recent years were reviewed. These burgeoning studies have been actively driving the innovative, and diversified development trends in the IOP monitoring area. However, several considerations should be given for designing or engineering the IOP transducer to avoid further potential risks that may be introduced into the eye by these wearable and implantable sensors.

### 4.1 Contact Lens

Contact lenses represent a kind of widely utilized device for vision correction, therapeutic and cosmetic purposes.^[^ ^179^ ^]^ To fit human eyes, the transparent commercial contact lenses were usually designed with a diameter of 13.5–14.5 mm, basic curve of 8.4–8.6 mm.^[^ ^180^ ^]^ In addition to the structure, oxygen transmissibility (Dk/t) is a critical parameter that represents the oxygen of corneal received to maintain its metabolic activity. Furthermore, wettability and excellent water content are another considerable factors correlating to the wearing comfortability. Therefore, the materials of polymerized hydroxyethyl methacrylate (pHEMA) and silicone hydrogel (SiH) were adopted for the fabrication of most commercial contact lenses. However, the volume and shapes of the contact lens‐based on these materials with higher water content are more vulnerable to hydration level,^[^ ^181^ ^]^ which will introduce undesirable noise for the IOP sensing element.^[^ ^109^, ^182^ ^]^

PET and rubbers are commonly adopted materials to form contact lens platform. Their fabrication process is scalable and relatively accessible.^[^ ^42^, ^183^, ^184^ ^]^ Moreover, the shapes of these materials‐based contact lenses are less susceptible to the variation of environmental hydration level. However, low oxygen transmissibility, unacceptable water content, and unexpected hardness result in poor comfortability and even potential risk (infections, allergies, vascular degenerative processes, and accidental damages) for cornea.^[^ ^185^ ^]^ While excellent interfacial modification process^[^ ^186^ ^]^ and specially patterned structures^[^ ^187^ ^]^ will provide potential approaches to accomplish hydrophilic surface and preferable oxygen transmissibility.

### 4.2 Conductance Materials

To the realization of flexibility and transmittance of the wearable and implantable IOP sensors, emerging nanomaterials such as Ag nanowires and carbon nanotubes offer promising candidates for flexible electronic elements fabrication. While the carbon nanotubes with unclear biosafety^[^ ^188^, ^189^ ^]^ should be carefully applied in wearable and especially implantable IOP sensors. Additionally, the Ag nanomaterials featured with excellent antimicrobial activities show potential application in anti‐virus as well as anti‐infectious wound dressings.^[^ ^190^ ^]^ However, researches exhibited that Ag nanomaterials were dissolved in the subcutaneous tissue and released silver ions rapidly. The dissociative silver ions also caused oxidative injury of bio‐tissue in the implanted site.^[^ ^191^ ^]^ Furthermore, Ag^+^ is capable of inactivating enzymes in forms of binding to thiol, amino, and carboxyl groups^[^ ^192^, ^193^, ^194^ ^]^ but also damage DNAs via formation of Ag^+^‐polynucleotide complexes.^[^ ^195^ ^]^ Moreover, silver products accumulated in eye will cause argyrosis.^[^ ^46^ ^]^

### 4.3 Structures

In addition to materials, more cautions should preferably be paid for several implants such the devices with cavity structure as capacitive, microfluidic and optical cavity‐based IOP sensors. These implants achieve IOP monitoring rely on the different pressure between inside and outside of cavity. Given the pain, risk, and additional cost that re‐operation could cause undesirable effects, implants need to exhibit long‐term biocompatibility, stability in eyeball. Therefore, perfect design and fabrication process are required to ensuring excellent encapsulation for the cavity structure. These measures could avoid aqueous humor drifting into the cavity, which enables durable properties for these passive implantable devices.

Furthermore, before invasive surgery meticulous cleaning for the implants should be carried out to avoid the introduction of bacteria or pathogens into anterior chamber, capsular bags, vitreous, and choroid. Because the presence of special physiological barriers (e.g., corneal epithelium, blood‐aqueous, and blood‐retinal barrier) of the human eye, efficient delivery of drugs into the eye is a great challenge.^[^ ^196^ ^]^ Therefore, any infection, inflammation that implants brought into the eye will cause unexpected misery for patients.

### 4.4 Experimental Model

Experimental models, which are quite important for assessing the performance and refining the design of wearable IOP transducer, have been utilized to advance the IOP sensors‐based on contact lens. The common models include porcine eye,^[^ ^102^, ^104^, ^109^, ^110^, ^111^, ^112^, ^132^, ^145^, ^146^, ^182^ ^]^ bovine eyeball,^[^ ^11^ ^]^ canine eye,^[^ ^113^ ^]^ rat's eyeball,^[^ ^36^ ^]^ rabbit eye,^[^ ^37^, ^103^, ^197^ ^]^ and artificial silicon eye model.^[^ ^42^, ^105^, ^145^, ^198^ ^]^ These models offer feasibility and convenience for the developments of wearable IOP detectors. While, there are critical differences in cornea parameters of this models such as thickness, curvature, and elastic modulus. These factors have an important effect on the accuracy of indirect IOP measurements that captures the significant biological signals by the means of detecting the cornea deformations.^[^ ^154^, ^199^ ^]^ Therefore, models with structure and mechanical property more similar or even identical to human eye should be employed, which could provide preferable opportunity to develop ideal wearable IOP sensors for glaucoma patients.

## 5 Future Prospects

To fabricate continuous IOP monitoring platform, inductive couple telemetry, piezoresistive, microfluidic, structural colors, and optical interference technologies were exploited. Meanwhile, promising materials, ingenious structures and clever fabrication process were adopted to achieve flexible, miniature devices that is highly suitable to be conveniently worn on cornea or micro‐invasively implanted in ocular. These wearable and implantable IOP sensors provide the access of continuous, long‐term and wireless IOP monitoring and avoid the temporal and spatial restrictions an inherent problem of clinical ophthalmotonometer. However, clinical applications of these new devices have been hindered by several unignored problems. And promising directions for the future research of IOP detectors are included here.

### 5.1 Structures

The iris, a critical ocular tissue, plays an important role in governing the amount of light reaching the retina.^[^ ^152^, ^200^ ^]^ The function of this tissue is beneficial for accomplishing an excellent quality of vision, avoiding individuals hypersensitive to light, and increasing the depth of focus.^[^ ^200^, ^201^ ^]^ In the area of implantable IOP sensors, the delicate ocular tissue surrounded by aqueous humor has been considered as an ideal site for install IOP sensors in anterior chamber.^[^ ^93^, ^156^, ^161^, ^162^, ^202^ ^]^ However, unfriendly installation of IOP sensors on iris will penetrate the delicate tissue. This operation will introduce damages in the dilator pupillae muscle and the sphincter pupillae muscle of the tender tissue. It will has a bad effect on the contractive and dilated function of the iris and even cause the traumatic iris deficiencies.^[^ ^203^ ^]^ This ocular disease can results in severe visual disability including glare, photophobia, and impaired visual function.^[^ ^201^, ^203^ ^]^ Therefore, proper consideration should be given for the fixed approaches of IOP sensors in anterior chamber. Anterior chamber intraocular lenses represent an typical implant placed in the anterior chamber of the eye to address the problems of cataract or myopia.^[^ ^204^, ^205^ ^]^ This mature ophthalmic platform may offers a promising candidate that load implantable IOP sensors to be positioned in anterior chamber. The idea may reduce the potential risk of long‐term implantation, and also minimize the complexity in surgery process.

### 5.2 Surface Modification

Posterior capsule opacification (PCO), the common complication following cataract surgery,^[^ ^206^ ^]^ derives from that residual lens epithelial cells undergo rapid proliferation and migration.^[^ ^207^, ^208^ ^]^ This fibrous metaplasia usually results in visual obscuration and even bring progressive loss of vision.^[^ ^209^, ^210^ ^]^ Notably, the incidence of this ocular disorder to five years after operation is nearly 100% for children and as well as 10–70% for adults.^[^ ^208^, ^211^ ^]^ Therefore, measures such as surface modification technologies of anti‐adhesion^[^ ^212^, ^213^ ^]^ should be employed to engineering the IOP transducer implanted in capsular bags.

### 5.3 Further Applications of IOP Detectors

Up to now, many efforts have demonstrated that raised IOP is correlated to several symptoms or disorders, such as diabetes,^[^ ^214^, ^215^ ^]^ hypertension,^[^ ^214^ ^]^ obesity,^[^ ^216^ ^]^ chronic kidney disease,^[^ ^71^ ^]^ Graves' ophthalmopathy,^[^ ^67^, ^68^, ^217^, ^218^ ^]^ and retinal vein ccclusion.^[^ ^219^ ^]^ However, the underlying mechanisms are still unclear and several critical relationships between the abnormal IOP and diseases remain controversial.^[^ ^59^, ^60^, ^61^, ^62^ ^]^ Therefore, the wearable and implantable IOP transducers have not been suitable for the applications of these disorders screening, diagnosis and even evaluations of therapeutic effect currently. Despite these considerable restrictions, sensors with the capabilities of continuous IOP gathering could provide invaluable data. These informations will pave the way for further exploring diseases' model and highly desirable prevention of complication. Beyond this, fluctuant IOP combined with many other messages recorded by various biosensors will endow researchers, clinicians with powerful insights to address the key scientific problems in the treatment of complex syndromes.

## 6 Conclusions

Overall, wearable and implantable IOP sensors possess great potential as continuous IOP monitoring platforms promoting earlier detection, accurate progression assessing and correct treatment scheme decision for glaucoma patients. It is conceived that the growing maturity of wearable and implantable IOP sensors and corresponding integrated systems would have the potential to provide preferable diagnosis technologies for glaucoma. Moreover, the promising devices could also be adopted to explore other disease's model and prevent undesirable complication.

## Conflict of Interest

The authors declare no conflict of interest.
